# Distinctive Tropical Forest Variants Have Unique Soil Microbial Communities, But Not Always Low Microbial Diversity

**DOI:** 10.3389/fmicb.2016.00376

**Published:** 2016-04-05

**Authors:** Binu M. Tripathi, Woojin Song, J. W. F. Slik, Rahayu S. Sukri, Salwana Jaafar, Ke Dong, Jonathan M. Adams

**Affiliations:** ^1^Department of Biological Science, College of Natural Sciences, Seoul National UniversitySeoul, South Korea; ^2^Seoul ZooSeoul, South Korea; ^3^Faculty of Science, Universiti Brunei DarussalamGadong, Brunei Darussalam

**Keywords:** biodiversity, microbial communities, soil pH, Southeast Asia, tropical rainforest

## Abstract

There has been little study of whether different variants of tropical rainforest have distinct soil microbial communities and levels of diversity. We compared bacterial and fungal community composition and diversity between primary mixed dipterocarp, secondary mixed dipterocarp, white sand heath, inland heath, and peat swamp forests in Brunei Darussalam, Northwest Borneo by analyzing Illumina Miseq sequence data of 16S rRNA gene and ITS1 region. We hypothesized that white sand heath, inland heath and peat swamp forests would show lower microbial diversity and relatively distinct microbial communities (compared to MDF primary and secondary forests) due to their distinctive environments. We found that soil properties together with bacterial and fungal communities varied significantly between forest types. Alpha and beta-diversity of bacteria was highest in secondary dipterocarp and white sand heath forests. Also, bacterial alpha diversity was strongly structured by pH, adding another instance of this widespread pattern in nature. The alpha diversity of fungi was equally high in all forest types except peat swamp forest, although fungal beta-diversity was highest in primary and secondary mixed dipterocarp forests. The relative abundance of ectomycorrhizal (EcM) fungi varied significantly between forest types, with highest relative abundance observed in MDF primary forest. Overall, our results suggest that the soil bacterial and fungal communities in these forest types are to a certain extent predictable and structured by soil properties, but that diversity is not determined by how distinctive the conditions are. This contrasts with the diversity patterns seen in rainforest trees, where distinctive soil conditions have consistently lower tree diversity.

## Introduction

Ecologists are fascinated by patterns in the biodiversity of tropical rainforests, including how and why communities differ from one another, and why diversity is greater in some places than others. In terms of known biodiversity, Southeast Asian tropical rainforests are one of the most diverse terrestrial ecosystems on Earth (Corlett, 2014), with numerous poorly studied habitat types within them. The major equatorial rainforest types in Southeast Asia are mixed dipterocarp forest (MDF), heath forest and peat swamp forest (Whitmore, 1984). Heath and peat swamp forests occur on acidic sandy and wet peaty soils, respectively, and support lower plant diversity compared to MDF (Bruenig and Droste, 1995; Davies and Becker, 1996; Slik et al., 2009). However, compared to macro-organisms, the community composition and diversity of microorganisms in these rainforest habitats is largely unknown.

Soil microorganisms constitute the largest proportion of the world’s biodiversity and are important to terrestrial ecosystem functioning (Prosser, 2012). Thus, understanding their biodiversity patterns and the major divers of these patterns in natural habitats may be important for prediction of ecosystem responses to a changing environment (Jing et al., 2015). Previous studies of tropical soils have indicated that land use changes in tropical regions influence soil microbial communities, which are mainly driven by changes in soil chemical properties (Jesus et al., 2009; Tripathi et al., 2012; Lee-Cruz et al., 2013; Rodrigues et al., 2013; Kerfahi et al., 2014). Soil pH is becoming recognized as one of the most import drivers of microbial community structure and diversity in tropical soils at various scales (Jesus et al., 2009; Tripathi et al., 2014). There have also been some studies which compared the soil microbial community composition and diversity in different rainforest habitat types (Satrio et al., 2009; Araujo et al., 2012; Miyashita et al., 2013; Pacchioni et al., 2014; Pupin and Nahas, 2014). However, most of these studies were concentrated in Neotropical regions.

The present study concentrates on variation in soil bacterial and fungal community composition and diversity in several different types of lowland tropical rainforest habitat within Brunei Darussalam, Northwest Borneo, in Southeast Asia. The MDF forests dominate the lowland forests of Borneo (Ashton, 1988; Slik et al., 2003, 2009), whereas tropical heath and peat swamp forests are relatively distinctive habitats compared to MDF forests in Borneo in terms of species composition and diversity (Brünig, 1974; MacKinnon, 1996; Cannon and Leighton, 2004). Though the above ground diversity is well-studied in these various rainforest types, it is still unclear whether different rainforest habitats have distinct microbial community composition and diversity, analogous to the distinct plant community composition and diversity levels of these habitats (Bruenig and Droste, 1995; Davies and Becker, 1996).

The present study was conducted in Brunei Darussalam, Northwest Borneo, in Southeast Asia. Across Brunei, the major rainforest types are MDF primary, MDF secondary and peat swamp forests, with smaller scattered areas of heath forests (Whitmore, 1984). This concentration of a range of different rainforest types in close proximity provides an opportunity to study the soil microbial community composition and diversity under common climatic conditions while also diminishing the potential effect of dispersal limitation, meaning that detected differences can most likely be ascribed to differences in the soil and plant community only. We used 16S rRNA gene and ITS1 region amplicon sequencing using Illumina MiSeq platform to address the following questions:

(1) How do different rainforest habitats influence the OTU composition of soil bacteria and fungi, and what are the major soil properties linked to bacterial and fungal community structure?
(2) What are the dominant higher level bacterial and fungal taxa in each rainforest habitat type, and how does their relative abundance vary with respect to different rainforest habitats?
(3) How does the alpha and beta-diversity of bacteria and fungi vary across different rainforest habitats?

We hypothesized that white sand heath, inland heath and peat swamp forests would show lower alpha- and beta-diversity of bacteria and fungi, with relatively distinct microbial communities compared to MDF primary and secondary forests due to distinctive conditions of these environments.

## Materials and Methods

### Site Description and Sample Collection

**FIGURE 1:**
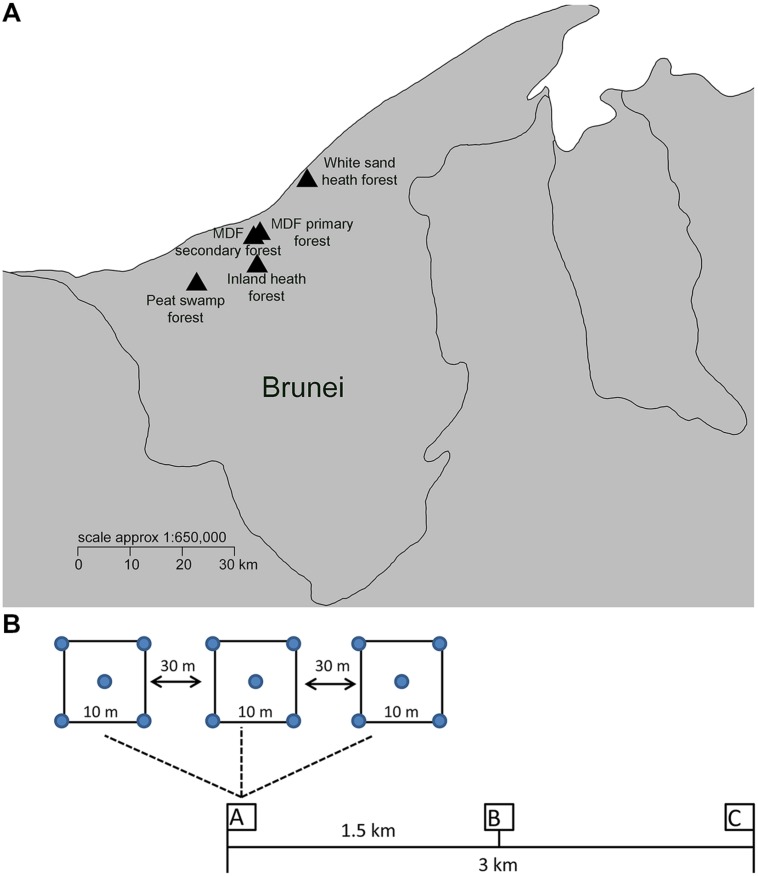
**(A)** Soil sample locations of different forest types in Brunei. **(B)** Sampling scheme, three clusters of samples (designated as A–C) were taken in each forest type within a 3 km transect. Within each cluster, three quadrats (10 m × 10 m in size) were collected at least 30 m apart along a smaller scale linear transect. Soil collected from the four corners and center of the each quadrat was pooled to make one samples for DNA extraction and soil property analysis.

Five different lowland tropical rainforest types in Brunei Darussalam, Northwest Borneo were selected for this study (**Figure 1**). These forest types were MDF primary, mixed dipterocarp secondary, white sand heath, inland heath, and peat swamp forests. The MDF primary forest is dominated by large tree species in the family Dipterocarpaceae and the forest structure is complex and multi-layered. The sampled MDF secondary forests were aged around 60 years (Davies and Becker, 1996). Previously, the secondary forest sampling area was covered with primary forest (Davies and Becker, 1996). MDF secondary forest is characterized by similar plant species composition to the MDF primary forest, but differing by the dominance of pioneer tree species such as *Macaranga*, *Vitex*, and *Dillenia* species. The secondary MDF is also has a more open structure, consisting of a complex mosaic of near- mature and regenerating forest patches with contrasting plant compositions and micro-climates. The white sand and inland heath forests differ considerably from MDF forest both in plant species and structure (having a low and uniform single-layered canopy with dense undergrowth full of shrubs, herbs, pitcher plants, etc.). The main difference between the two heath forest types we sampled is that inland heath forest has low drainage capacity compared to white sand heath forest, which means that the white sand heath forest is being more susceptible to drought, while the inland heath forest can be flooded for part of the year. The peat swamp forest sampled in this study is dominated by a single canopy species of even aged/sized trees of *Shorea albida* (Dipterocarpaceae), while general plant diversity is much lower than in MDF, although the overall forest structure can be quite similar.

Field sampling was carried out during the month of June 2014, during a time with characteristic climate conditions in which afternoon rainstorms occurred about every other day (Becker, 1992). Brunei has a seasonal climate, with two drier periods February/March and July/August (Becker, 1992), and a mean annual rainfall above 2300 mm (David and Sidup, 1996). Three clusters of samples were taken in each forest type within a 3 km transect (**Figure 1**). Within each cluster, three quadrats (10 m × 10 m in size) were collected at least 30 m apart along a smaller scale linear transect (**Figure 1**). Each individual sample consisted of five pooled samples (each approximately 50 g from the four corners and one center point of the quadrat). The top 10 cm of soil was collected in a sterile sampling bag after removing the litter layer. A total of 45 samples were collected from five different forest types (nine samples from each forest type). The collected soil samples were homogenized by sieving (2 mm sieve), and stored at -20°C until DNA extraction.

### Soil Properties Analysis

Geographical co-ordinates and soil temperature at 5 cm depth were measured using a GPS device and a soil thermometer at each sampling quadrat during field sampling. Soil pH, gravimetric water content, organic matter content, total nitrogen and available phosphorus concentrations, and soil texture were measured at Universiti Brunei Darussalam using the standard methods (Allen, 1989). Total nitrogen content was determined by Kjeldahl method. Soil available phosphorus was extracted using Bray’s reagent (0.025 M hydrochloric acid and 0.03 M ammonium fluoride), and the phosphorus concentration in the extracts was then determined using a UV-spectrophotometer (UV-1800, Shimadzu, Kyoto, Japan). Soil organic matter content was determined after incineration in a muﬄe furnace at 550°C for 2 h, according to the methodology described by Allen (1989).

### DNA Extraction, PCR, and Illumina Sequencing of 16S rRNA Gene and ITS1 Region

Soil DNA was extracted from each of the collected samples using the PowerSoil DNA extraction kit (MO BIO Laboratories, Carlsbad, CA, USA) following manufacturer’s instructions, and DNA samples were sent to Macrogen Incorporated (Seoul, Korea) for PCR amplification and sequencing. The extracted DNA samples were amplified for V3 and V4 region of 16S rRNA gene using the primer pairs Bakt_341F (5′-CCTACGGGNGGCWGCAG-3′) and Bakt_805R (5′-GACTACHVGGGTATCTAATCC-3′) for characterizing the bacterial communities (Herlemann et al., 2011). The fungal internal transcribed spacer (ITS) region 1 was amplified using ITS1F (5′-CTTGGTCATTTAGAGGAAGTAA-3′) and ITS2 (5′-GCTGCGTTCTTCATCGATGC-3′) primer pairs (White et al., 1990; Gardes and Bruns, 1993). The resulting 16S rRNA gene and ITS1 amplicons were sequenced using paired-end (2 × 300 nt) Illumina Miseq system (Illumina, USA).

### Sequence Processing

The paired-end sequences of 16S rRNA gene and ITS1 amplicons were assembled using PANDAseq assembler (Masella et al., 2012). The initial sequence processing steps such as quality filtering and sequence alignment were performed using mothur (Schloss et al., 2009). The 16S rRNA gene sequences were aligned against a SILVA alignment^1^ Chimeric 16S rRNA gene and ITS1 sequences were identified using ‘chimera.uchime’ command implemented in mothur in *de novo* mode (Edgar et al., 2011), and removed. Taxonomic assignments of all the high quality 16S rRNA gene and ITS1 sequences were performed in mothur using the EzTaxon-e database^2^ (Kim et al., 2012), and UNITE database (Abarenkov et al., 2010), respectively. To determine the ectomycorrhizal (EcM) fungi we matched the fungal taxonomic assignments with known EcM lineages (Tedersoo et al., 2010). The operational taxonomic units (OTUs) were assigned for 16S rRNA gene and ITS 1 sequences using mothur and QIIME implementation of UCLUST (Caporaso et al., 2010; Edgar, 2010), respectively, with a threshold of ≥97% sequence similarity. The entire singleton OTUs were removed prior to analysis. All the 16S rRNA gene and ITS1 sequences used in this study are deposited to metagenomic-RAST server (Meyer et al., 2008) under the project ID 14875^3^

### Statistical Analysis

Prior to statistical analysis, a random subset of 3,352 and 4,207 sequences per sample was generated for 16S rRNA gene and ITS 1 sequences, respectively, to correct for the differences in number of reads. To assess the differences in soil properties among different forest types, we used analysis of variance (ANOVA) or Kruskal–Wallis tests for normal and non-normal data, respectively. Furthermore, parametric (Tukey’s HSD test) or non-parametric (pairwise Wilcox test) *post hoc* tests were used in case of significant results of ANOVA or Kruskal–Wallis tests, respectively. We used the Benjamini-Hochberg correction to assess pairwise comparisons (*P* < 0.05; Benjamini and Hochberg, 1995). A principal components analysis (PCA) was performed on the correlation matrix of soil properties data of each sample in Canoco 5.0 (Biometrics, Wageningen, The Netherlands). We used permutational multivariate analysis of variance (PerMANOVA, ‘adonis’ function in vegan R package) to test the effect of forest type on a Euclidean distance matrix of normalized soil properties data with 9999 random permutations.

Cluster analysis was performed on Bray–Curtis distance matrices of bacterial and fungal OTUs by using an unweighted pair group mean (UPGMA) algorithm implemented in the ‘hclust’ function of vegan R package (Oksanen et al., 2007). As 16S rRNA genes are suitable for phylogenetic analysis, a unweighted UniFrac distance matrix was also generated for bacteria (Lozupone et al., 2011). Bray–Curtis and unweighted UniFrac distance matrices were further visualized by non-metric multidimensional scaling (NMDS) plots. Furthermore, PerMANOVA was used to evaluate the effect of forest type on Bray–Curtis and unweighted UniFrac distance matrices with 9999 random permutations. To detect possible associations between bacterial and fungal community structure and soil properties, the vectors of significant soil properties (*P* < 0.05) were fitted onto ordination space using the ‘envfit’ function of the vegan R package with 999 random permutations.

The significant differences in composition and diversity of bacterial and fungal taxa in different forest types were analyzed by ANOVA or Kruskal–Wallis tests as described above. To test the relationship between soil properties and the relative abundance of dominant bacterial and fungal phyla, we used the Spearman rank correlation test. We performed linear regression analysis to test for differences in alpha-diversity (Shannon index) in relation to soil properties. We used the betadisper function of ‘vegan’ R package to assess the differences in beta-diversity among different forest types, and significance of this test was determined using 999 permutations.

## Results

### Soil Properties among Forest Types

All the measured soil properties varied significantly among different forest types, except for total nitrogen and silt concentrations (**Table 1**). PCA of the different soil properties measured indicated that peat swamp forest sites were clearly distinct from other forest types (Supplementary Figure S1); however, sites from other forest types were not well-separated from each other (Supplementary Figure S1). The first two axis of the PCA explained about 71% of the total variance, with axis 1 and 2 explaining 51.6 and 19.4% of the total variance, respectively. The PerMANOVA analysis revealed a statistically significant effect of forest type on soil properties (*P* < 0.001, 9999 permutations).

**Table 1:** Values of (mean ± SD) of soil properties in different forest types.

Soil properties	MDF primary forest	MDF secondary forest	White sand heath forest	Inland heath forest	Peat swamp forest
Temperature (°C)^2^	26.1 ± 0.4 b	27.8 ± 1.5 a	27.4 ± 0.7 a	26.2 ± 0.4 b	26.7 ± 0.3 a
Gravimetric water content (gg^-1^)^2^	0.24 ± 0.06 c	0.18 ± 0.02 c	0.26 ± 0.13 c	0.46 ± 0.14 b	2.95 ± 0.53 a
pH^1^	3.9 ± 0.2 b	4.3 ± 0.3 a	4.2 ± 0.3 ab	3.2 ± 0.2 c	3.4 ± 0.2 c
Organic matter (%)^2^	12.6 ± 3.5 c	10.4 ± 5.9 c	46.7 ± 11.9 b	45.1 ± 17.7 b	97.7 ± 0.6 a
Total nitrogen (mg g^-1^)^1^	16.1 ± 4.3 a	10.2 ± 5.5 a	17.2 ± 9.7 a	15.1 ± 13.2 a	9.2 ± 1.6 a
Available phosphorus (μg g^-1^)^2^	67.6 ± 2.8 b	68.5 ± 8.1 b	64.4 ± 2.6 b	64.1 ± 2.5 b	85.4 ± 7.7 a
Clay (%)^2^	7.9 ± 5.7 a	4.9 ± 2.5 ab	2.4 ± 2.3 bc	2.2 ± 0.9 c	11.6 ± 9.2 a
Silt (%)^2^	71.7 ± 6.3 a	75.3 ± 3.6 a	72.8 ± 3.5 a	73.6 ± 4.7 a	70.1 ± 9.9 a
Sand (%)^2^	20.4 ± 2.4 ab	19.8 ± 2.0 ab	24.7 ± 4.3 a	24.2 ± 5.3 a	18.1 ± 1.8 b

*Means on the same row with different letters were significantly different (*P <* 0.05) based on Tukey’s HSD test (1) or pairwise Wilcox test (2) followed by Benjamini-Hochberg correction for multiple comparisons*.

### Bacterial and Fungal Community Composition among Forest Types

**FIGURE 2:**
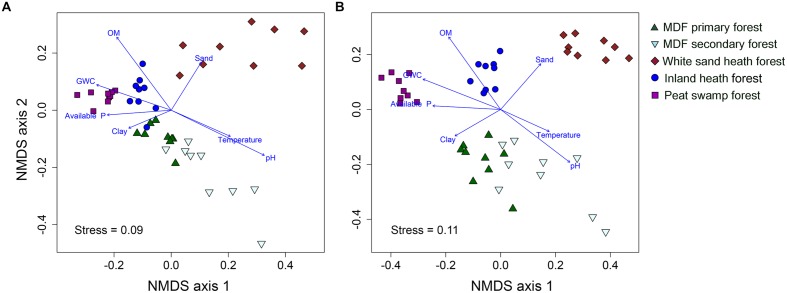
**Non-metric multidimensional scaling plot of **(A)** bacterial, and **(B)** fungal communities based on pairwise Bray–Curtis distances**. A vector overlay of the significantly correlated variables is shown on the plot. GWC, gravimetric water content and OM, organic matter content.

The UPGMA clustering analysis based on Bray–Curtis distance showed that bacterial and fungal community compositions were largely separated by forest types (Supplementary Figure S2). The bacterial and fungal communities in white sand heath forest were most distinct from all other forest types (Supplementary Figure S2). Whereas, MDF primary and secondary forests had most similar bacterial and fungal community composition. However, in the MDF secondary forest there were two and four extreme bacterial and fungal communities, respectively. The bacterial communities of inland heath and peat swamp forests were at the same distance level to the MDF primary and secondary forest communities, whereas, bacterial communities in these forests were in turn at the same distance level to those in the white sand heath forest. In the case of fungi, inland heath forest communities are closer in composition to MDF forests than to peat swamp and white sand heath forests communities. The UPGMA clustering results were further corroborated by the NMDS ordination plot, which also showed that bacterial and fungal community compositions were segregated by forest type (**Figures 2A,B**). The PerMANOVA analyses indicated that forest type explained 36.1 and 37.8% variation in bacterial and fungal community composition, respectively (*P* < 0.001, 9999 permutations). The phylogenetic community composition of bacteria, based on unweighted UniFrac distance also displayed similar pattern as that of bacterial OTU composition (Supplementary Figure S3), and also significantly influenced by forest type (PerMANOVA, *P* < 0.001, 9999 permutations).

To further investigate the effect of soil properties on bacterial and fungal community structure, the vectors of environmental variables were fitted onto ordination space. The environmental fitting analysis indicated that of the measured soil properties, soil pH, organic matter content, gravimetric water content, available phosphorus, temperature, sand and clay content were strongly correlated with bacterial and fungal community structure (Supplementary Figure S3 and **Figures 4A,B**).

### Dominant Bacterial and Fungal Taxa

**FIGURE 3:**
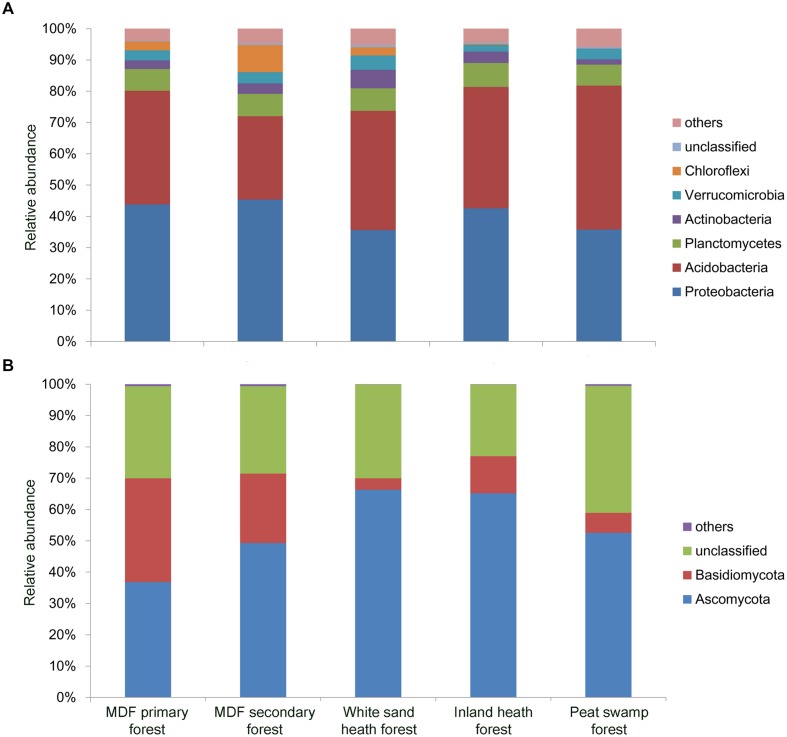
**Relative abundance of dominant **(A)** bacterial phyla observed in 16S rRNA gene sequences and **(B)** fungal phyla in ITS1 sequences in different forest types**.

A total of 150,840 good quality bacterial 16S rRNA gene sequences were obtained (3,352 randomly selected reads per sample). *Proteobacteria* was the most dominant bacterial phylum (40.6% of all bacterial sequences) followed by *Acidobacteria* (37.2%), *Planctomycetes* (7.1%), *Actinobacteria* (3.5%), *Verrucomicrobia* (3.4%), and *Chloroflexi* (2.9%; **Figure 3A**). Except *Planctomycetes*, the relative abundance of these phyla varied significantly (*P* < 0.05) among forest types (**Table 2**). For fungal ITS1 sequences, a total of 189,315 high quality sequences were obtained from 45 samples (4,207 randomly selected reads per sample). The most abundant fungal phylum detected across all samples was Ascomycota (54.1% of all fungal sequences) followed by Basidiomycota (15.4%), and 30.1% of the detected sequences were unclassified (**Figure 3B**). The relative abundance of these most abundant fungal phyla varied significantly in relation to different forest types (**Table 2**). The relative abundance of Ascomycota was higher in white sand and inland heath forests (**Table 2**), whereas the relative abundance of Basidiomycota was higher in MDF primary and secondary forests (**Table 2**).

**Table 2:** Comparison of relative abundance (mean ± SD) of dominant bacterial and fungal phyla, and EcM fungi among forest types.

Dominant taxa	MDF primary forest	MDF secondary forest	White sand heath forest	Inland heath forest	Peat swamp forest
**Bacteria**					
*Proteobacteria*^1^	43.8 ± 3.9 a	45.4 ± 5.5 a	35.6 ± 3.3 b	42.5 ± 4.3 a	35.8 ± 2.0 b
*Acidobacteria*^1^	36.3 ± 5.0 b	26.6 ± 4.3 c	38.1 ± 5.0 b	38.8 ± 6.7 b	46.0 ± 3.3 a
*Planctomycetes*^1^	7.0 ± 0.9 a	7.1 ± 1.5 a	7.2 ± 1.0 a	7.7 ± 1.5 a	6.7 ± 1.0 a
*Actinobacteria*^2^	2.8 ± 1.0 b	3.4 ± 1.5 b	5.9 ± 2.6 a	3.6 ± 2.1 b	1.7 ± 0.9 c
*Verrucomicrobia*^2^	3.2 ± 0.5 a	3.6 ± 1.5 a	4.6 ± 2.3 a	2.2 ± 0.5 b	3.5 ± 0.8 a
*Chloroflexi*^2^	2.9 ± 1.8 b	8.7 ± 6.1 a	2.6 ± 2.4 b	0.4 ± 0.5 c	0.1 ± 0.1 c
**Fungi**					
Ascomycota^1^	36.9 ± 18.1 b	49.3 ± 18.7 ab	66.3 ± 12.4 a	65.2 ± 16.6 a	52.6 ± 23.8 ab
Basidiomycota^1^	33.1 ± 17.7 a	22.2 ± 17.6 ab	3.7 ± 2.9 c	11.8 ± 11.8 bc	6.4 ± 3.9 bc
**EcM fungi**^2^	25.8 ± 18.0 a	12.8 ± 17.5 abc	0.1 ± 0.0 d	9.2 ± 11.1 b	0.9 ± 0.8 c

*Different letters represent means that were significant different (*P <* 0.05) based on Tukey’s HSD test (1) or pairwise Wilcox test (2) followed by Benjamini-Hochberg correction for multiple comparisons*.

A total of 18,460 sequences belonged to known EcM fungal genera, representing around 9.7% of the total detected fungal sequences. The relative abundance of the detected EcM fungal genera varied significantly between forest types, with highest and lowest relative abundance observed in MDF primary forest and white sand heath forest, respectively (*P* < 0.0001; **Table 2**). The most abundant EcM fungal genus was *Russula* (78% of total EcM sequences), followed by *Amanita*, *Thelephora*, and *Tomentella*. The relative abundance of *Russula* also varied significantly between forest types, and showed similar pattern to that of total EcM fungi (Supplementary Table S1).

The relative abundance of *Proteobacteria*, *Acidobacteria*, and *Chloroflexi* was significantly correlated with gravimetric water content, soil pH, and organic matter content (**Table 3**). Whereas, the reactive abundance of *Planctomycetes* and *Actinobacteria* correlated with clay content (**Table 3**). Additionally, soil temperature and sand content was also found to be correlated with the relative abundance of *Acidobacteria* and *Actinobacteria*, respectively. The relative abundance of *Verrucomicrobia* was not correlated with any of the environmental variables measured (**Table 3**). The relative abundance of the most dominant fungal phylum Ascomycota was significantly correlated only with clay content, whereas the relative abundance of Basidiomycota the other dominant fungal phylum was significantly correlated with gravimetric water content and organic matter content.

**Table 3:** Spearman rank correlations between soil properties and the relative abundance of dominant bacterial and fungal phyla, and alpha diversity indices.

Jamini	Temperature (°C)	GWC (gg^-1^)	pH	Organic matter (%)	Total nitrogen (mg g^-1^)	Available phosphorus (μg g^-1^)	Clay (%)	Sand (%)	Silt (%)
**Bactetial phyla**								
*Proteobacteria*	0.04	-0.6***	0.52***	-0.58***	0.24	-0.11	0.1	0.08	-0.03
*Acidobacteria*	-0.34*	0.61***	-0.66***	0.65***	-0.2	0.26	0.08	-0.11	-0.15
*Planctomycetes*	0.04	-0.14	0.23	0.04	0.26	-0.05	-0.44**	0.16	0.18
*Actinobacteria*	0.12	-0.21	0.11	-0.13	0.26	-0.59	-0.31*	0.38**	0.09
*Verrucomicrobia*	0.08	0.21	0.02	0.09	0.11	0.04	0.26	-0.2	-0.02
*Chloroflexi*	0.09	-0.52***	0.51***	-0.71***	0.17	-0.28	0.01	0.11	0.09
**Fungal phyla**								
*Ascomycota*	0.08	0.19	-0.11	0.3	-0.05	-0.21	-0.37*	0.26	0.1
*Basidiomycota*	-0.09	-0.44**	0.27	-0.51***	0.03	0.03	0.29	-0.09	-0.12
**Alpha diversity bacteria**									
OTU richness	0.49**	-0.45**	0.8***	-0.45**	0.12	-0.05	0.04	-0.07	0.18
Shannon index	0.51***	-0.45**	0.8***	-0.48**	0.11	-0.02	0.1	-0.07	0.19
**Alpha diversity fungi**									
OTU richness	-0.3	-0.28	0.13	-0.4*	0.15	-0.41**	-0.18	0.26	0.14
Shannon index	0.03	-0.25	0.25	-0.24	-0.03	-0.43**	-0.35*	0.44**	0.12

*^∗^*P* < 0.05; ^∗∗^*P* < 0.01; ^∗∗∗^*P* < 0.001*.

### The Alpha and Beta-Diversity of Bacteria and Fungi

**FIGURE 4:**
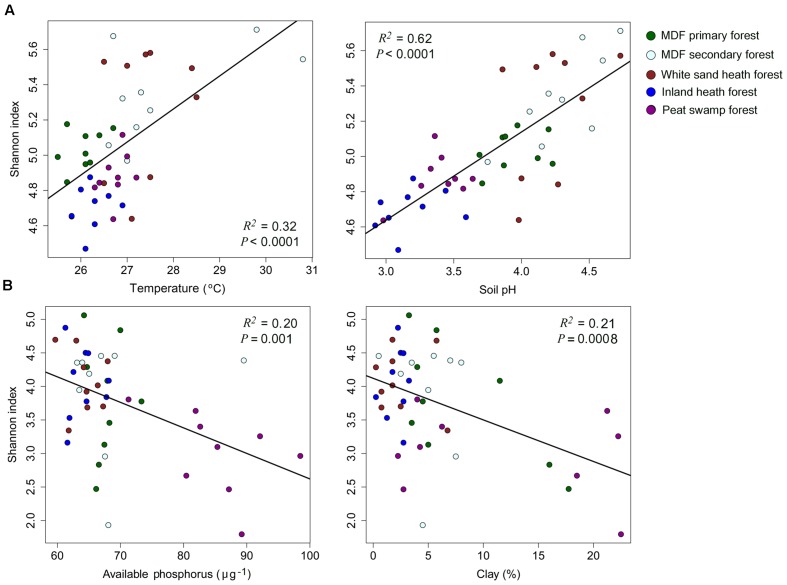
**The relationships between **(A)** bacterial, and **(B)** fungal Shannon index and soil properties with symbols coded by forest types**. Linear regressions were used to test the correlation between Shannon index and soil properties.

**FIGURE 5:**
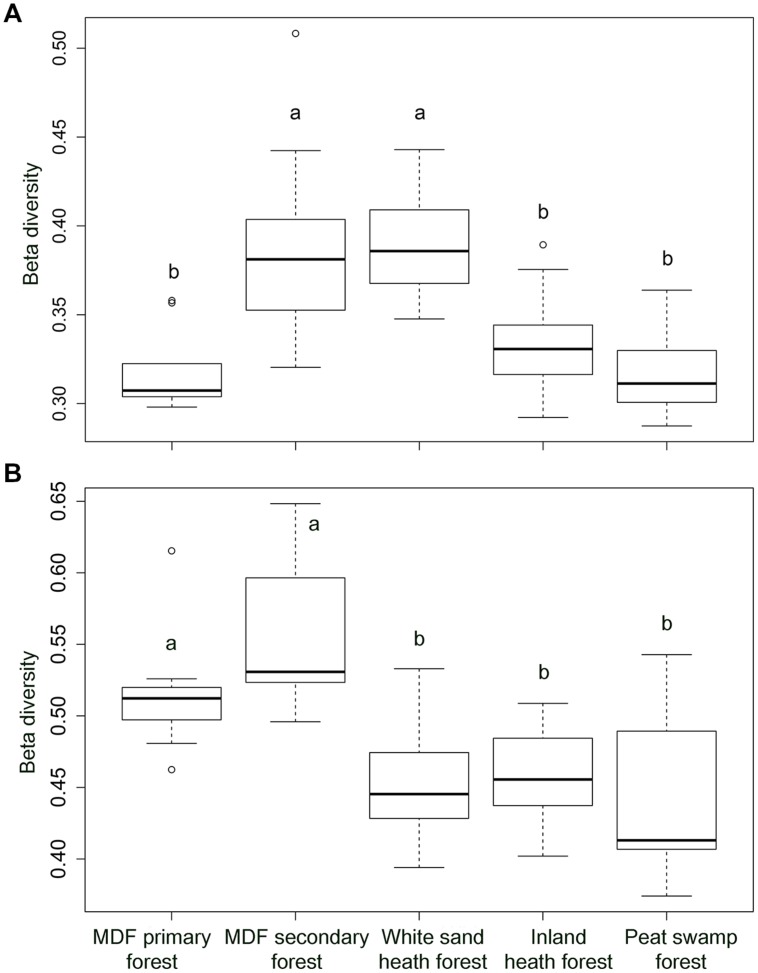
**The Whittaker beta-diversity of **(A)** bacterial community and **(B)** fungal community in five different forest types in Brunei, Borneo**. Significant differences (*P* < 0.05) between forest types, when present, are indicated by different letters.

The alpha-diversity index (OTU richness and Shannon index) of both bacteria and fungi also varied significantly among forest types (**Table 4**). The lowest average bacterial alpha diversity was observed in inland heath and peat swamp forest, whereas fungal alpha diversity was lowest in peat swamp forest only, although due to high variation in diversity values, considerable overlap in diversity existed between some forest types (**Table 4**). Bacterial Shannon diversity index correlated positively with soil temperature and pH (**Figure 4A**), whereas gravimetric water content and organic matter content of the soils displayed negative correlation with bacterial diversity indices (**Table 3**). Available phosphorus and soil clay content were negatively correlated with the Shannon index of the fungi (**Figure 4B**), while sand content was found to be positively correlated with fungal Shannon index (**Table 3**). The Whittaker beta-diversity of bacterial and fungal communities, measured as the average distance of all samples to the centroid in each forest type varied significantly among forest types (**Figure 5**). The MDF secondary and white sand heath forests having highest bacterial beta-diversity, whereas MDF primary and secondary forests had highest fungal beta-diversity (**Figure 5**).

**Table 4:** The alpha diversity indices (OTU richness and Shannon index) of bacteria and fungi in different forest types.

	MDF primary forest	MDF secondary forest	White sand heath forest	Inland heath forest	Peat swamp forest
**Bacteria**					
OTU richness	619 ± 37 ab	703 ± 82 a	688 ± 93 a	510 ± 46 c	567 ± 44 bc
Shannon index	5.0 ± 0.1 bc	5.3 ± 0.3 a	5.3 ± 0.4 ab	4.7 ± 0.1 d	4.9 ± 0.1 cd
**Fungi**					
OTU richness	390 ± 94 a	361 ± 80 a	318 ± 55 ab	372 ± 73 a	243 ± 44 b
Shannon index	3.8 ± 0.9 ab	3.9 ± 0.9 ab	4.1 ± 0.5 a	4.1 ± 0.5 a	3.0 ± 0.6 b

*Values with different letters were significantly different (*P* < 0.05) based on Tukey’s HSD test followed by Benjamini-Hochberg correction for multiple comparisons*.

## Discussion

### Distinct Soil Conditions amongst the Different Forest Types

The soil conditions varied significantly among different forest types (**Table 1**), reflecting the broad scale mosaic of environments within the lowland forests of Brunei (Moran et al., 2000; Din et al., 2015). The primary dipterocarp forest was toward the more acidic end of the normal range of pH for lowland terra firme rainforest (about pH 3.7–5.5), with typical available P and total N content (Sukri et al., 2012). The secondary forest had somewhat higher pH, but similar available P and total N levels. Soil temperature in the secondary forest at the time of sampling was somewhat higher on average, likely due to the more open canopy allowing greater daytime heating of the soil surface. Organic matter content was higher in both white sand and inland heath forests compared to MDF primary and secondary forest. The water logged environment of heath forests with limited oxygen levels might lead to accumulation of organic matter content (Moran et al., 2000). The sand content of both heath forest types was similar to the MDF primary and secondary forests. Our own examination of these soils before analyses showed that the white quartz grain component of the soils in both these heath forest types was unusually fine grade at these sites, and probably it ended up classified as silt grade. The inland heath forest was very acidic and high in soil gravimetric water content, showing swampy conditions. Swampy areas in heath forests are quite common in this region (Moran et al., 2000; Din et al., 2015). The peat swamp forest soils were very acidic, with higher organic matter and available P contents than the other forest types, and its gravimetric water content was much higher – reflecting the high water table and the abundant spongy peat. The higher level of nutrients in peat swamp forest is not unusual, as the peat soil is generated from the accumulation of partially decayed organic matter due to water logged conditions with limited oxygen supply (Andriesse, 1988; Satrio et al., 2009).

### Are There Distinct Microbial Communities on Different Rainforest Types?

The results of our comparison of soil bacterial and fungal communities across Brunei forests revealed that there are distinct community types in different types of rainforest. The community composition of fungi and bacteria in each forest type was significantly different from all of the others, but the most distinct community is that of the peat swamp forest. The distinct nature of these communities suggests that there is strong environmental and perhaps evolutionary selection for both bacterial and fungal OTUs better adapted in each environment. The clustering patterns of bacterial and fungal communities were very similar to one another, and influenced by soil pH, organic matter content, gravimetric water content, available phosphorus, temperature, sand and clay content. Previous studies on tropical soils have also shown that microbial community composition is influenced by variations in underlying soil properties due to land use change (Jesus et al., 2009; Tripathi et al., 2012, 2013; Lee-Cruz et al., 2013; Kerfahi et al., 2014).

The most abundant bacterial phyla detected across the samples were *Proteobacteria* and *Acidobacteria*, which is consistent with the results of previous studies on rainforest soils (Kanokratana et al., 2011; Tripathi et al., 2012; Lee-Cruz et al., 2013). The relative abundance of *Proteobacteria* was significantly lower in white sand heath and peat swamp forests, and this result could be due to restricted nutrient availability in oxygen-limited waterlogged environments of white sand heath peat swamp forests (Moran et al., 2000; Page et al., 2006). It has been shown that the relative abundance of major proteobacterial subphyla increases with nutrient additions (Leff et al., 2015). However, the relative abundance of *Acidobacteria* was significantly higher in peat swamp forests and negatively correlated with soil pH – a result which is to be expected as peat soils had very low pH, and most of the acidobacterial lineages are shown to dominate acidic soil environments (Jones et al., 2009). The relative abundance of *Actinobacteria* was highest in white sand heath forest, and positively correlated with the sand content of soil. These results are in agreement with previous observations that the members of phylum *Actinobacteria* are generally abundant in sandy forest soils (Russo et al., 2012; Pacchioni et al., 2014). The dominance of photosynthetic bacterial phylum *Chloroflexi* in secondary forests could be explained by that, due to more open canopy secondary forest soils are exposed to sunlight to a greater extent than other forest soils (Nacke et al., 2014).

At the broad taxonomic level, the relative abundance of fungal taxa detected in this study is similar to the soils of other tropical regions, where Ascomycota and Basidiomycota are also the most predominant phyla (Kerfahi et al., 2014; McGuire et al., 2014). Compared to MDF primary forest, there is an increased proportion of Ascomycota in other forest types. Ascomycota are often found at higher abundance in stressful environments (De Beeck et al., 2015), and the communities here appears to reflect this pattern. However, the lower relative abundance of Basidiomycota in white sand heath forest, inland heath forest and peat swamp forest reflect the distinctive conditions in these forest types compared to MDF forests, as numerous Basidiomycota fungi tend to be slow-growing, late-successional fungi that are sensitive to physical and chemical perturbations (Frankland, 1998; Osono, 2007).

### Are Distinctive Conditions Associated with Lower Fungal and Bacterial Diversity?

We expected to find lower alpha- and beta-diversity of bacteria and fungi in the more distinctive environments of the heath and peat swamp forests. This would be due to a combination of the low likelihood of lineages acquiring the evolutionary adaptations necessary to live in the conditions of low pH and water logged environments with limited oxygen supply. However, the observed diversity patterns did not follow these predictions. Alpha diversity of bacteria was higher in the white sand heath forest than in MDF primary forest, and similar to MDF secondary forest, while inland heath and peat swamp forest had almost similar level of alpha diversity to the MDF primary forest. When samples across all the forest types were compared in relation to soil parameters, pH emerged as overwhelmingly the strongest predictor of bacterial alpha diversity (**Figure 4A**). This result gives further confirmation of the generality of the pattern observed in other contexts around the world (Fierer and Jackson, 2006; Lauber et al., 2009; Tripathi et al., 2012), that bacterial alpha diversity increases toward neutral pH. Bacterial beta-diversity was highest in the white sand heath forest and in the MDF secondary forest – the same pattern as for bacterial alpha diversity. The other forest types had almost similar levels of bacterial beta diversity to one another. It appears that in this case tree species diversity has no bearing on the beta-diversity, perhaps reflecting the generally looser relationships between soil bacterial diversity and particular tree hosts (Millard and Singh, 2010).

Fungal alpha diversity was the same in all of the forest types except peat swamp forest. Thus, despite the apparently extreme conditions of two types of heath forests, fungal alpha diversity is no lower than in MDF primary or secondary forests. Only the peat swamp forest, perhaps because of waterlogged conditions, had lower fungal diversity. Beta diversity of fungi was, however, greater in the MDF primary and secondary forests than in the heath and peat swamp forests. This might be explicable in terms of the lower plant species diversity of these other non-terra firme forest types (Davies and Becker, 1996). Fungi often are involved in direct interactions with plants (Broeckling et al., 2008; Millard and Singh, 2010), and mycorrhizal fungi are specialized to grow under direct symbiotic relationships with plants (Gao et al., 2013). The greater beta diversity in the two terra firme forest types might then reflect the greater tree species diversity of these, with different samples able to reflect the range of host-tree-specific fungal communities that are present. An important role of the woody plant cover is also supported by the dominance of EcM fungi in MDF forests, EcM fungal groups are often dominant in Southeast Asian dipterocarp forests (Peay et al., 2010; Brearley, 2012; McGuire et al., 2014). Also importance might be a greater range of different saprotrophic fungal communities resulting from the input of different litter types from a more diverse assemblage of tree species.

Overall, this study confirmed our expectations that within the tropical rainforest, there is a strong degree of ecological differentiation in soil bacterial and fungal communities. However, the patterns in soil microbial diversity that we found amongst the various forest types in Brunei do not closely conform to our predictions that distinctive environments would show lower alpha- and beta-diversity of bacteria and fungi. There is need for further theoretical consideration to try to explain why the apparently distinctive and geologically ‘ephemeral’ environments of heath and peat swamp forests are about as diverse, or more diverse, than terra firme MDF primary and secondary forests.

## Author Contributions

BT, JS, RS, and JA designed the study, BT, WS, JS, RS, and SJ completed fieldwork in Brunei, BT, WS, and SJ processed samples in the laboratory, BT and KD completed data processing and analysis, BT and JA produced the first draft of the manuscript, and all authors edited the manuscript.

## Conflict of Interest Statement

The authors declare that the research was conducted in the absence of any commercial or financial relationships that could be construed as a potential conflict of interest.

## Footnotes

1 http://www.arb-silva.de/.

2 http://eztaxon-e.ezbiocloud.net/

3 http://metagenomics.anl.gov/linkin.cgi?project=14875.

## References

[B1] Abarenkov K. Henrik Nilsson R. Larsson K. H. Alexander I. J. Eberhardt U. Erland S. (2010). The UNITE database for molecular identification of fungi–recent updates and future perspectives. *New Phytol.* 186 281–285. 10.1111/j.1469-8137.2009.03160.x 20409185

[B2] Allen S. E. (1989). *Chemical Analysis of Ecological Materials.* Oxford: Blackwell Scientific Publications.

[B3] Andriesse J. (1988). *Nature and Management of Tropical Peat Soils.* Rome: Food and Agriculture Organization of the United Nations Soils Bulletin 59 165.

[B4] Araujo J. F. de Castro A. P. Costa M. M. Togawa R. C. Júnior G. J. P. Quirino B. F. (2012). Characterization of soil bacterial assemblies in Brazilian savanna-like vegetation reveals acidobacteria dominance. *Microb. Ecol.* 64 760–770. 22570118 10.1007/s00248-012-0057-3

[B5] Ashton P. S. (1988). Dipterocarp biology as a window to the understanding of tropical forest structure. *Ann. Rev. Ecol. Syst.* 19 347–370. 10.1146/annurev.es.19.110188.002023

[B6] Becker P. (1992). Seasonality of rainfall and drought in Brunei Darussalam. *Brunei Mus. J.* 7 99–109.

[B7] Benjamini Y. Hochberg Y. (1995). Controlling the false discovery rate: a practical and powerful approach to multiple testing. *J. R. Stat. Soc. Series B Stat. Methodol.* 57 289–300.

[B8] Brearley F. Q. (2012). Ectomycorrhizal associations of the Dipterocarpaceae. *Biotropica* 44 637–648. 10.1111/j.1744-7429.2012.00862.x

[B9] Broeckling C. D. Broz A. K. Bergelson J. Manter D. K. Vivanco J. M. (2008). Root exudates regulate soil fungal community composition and diversity. *Appl. Environ. Microbiol.* 74 738–744. 10.1128/AEM.02188-07 18083870 PMC2227741

[B10] Bruenig E. Droste H. (1995). “Structure, dynamics and management of rainforests on nutrient-deficient soils in Sarawak,” in *Ecology, Conservation, and Management of Southeast Asian rainforests*, eds Primack R. B. Lovejoy T. E. (London: Yale University Press), 41–53.

[B11] Brünig E. F. (1974). *Ecological Studies in the Kerangas Forests of Sarawak and Brunei.* Kuching: Borneo Literature Bureau for Sarawak Forest Department.

[B12] Cannon C. H. Leighton M. (2004). Tree species distributions across five habitats in a Bornean rain forest. *J. Veg. Sci.* 15 257–266. 10.1111/j.1654-1103.2004.tb02260.x

[B13] Caporaso J. G. Kuczynski J. Stombaugh J. Bittinger K. Bushman F. D. Costello E. K. (2010). QIIME allows analysis of high-throughput community sequencing data. *Nat. Methods* 7 335–336. 10.1038/nmeth.f.303 20383131 PMC3156573

[B14] Corlett R. T. (2014). *The Ecology of Tropical East Asia.* Oxford: Oxford University Press.

[B15] David A. Sidup S. (1996). *Brunei Metereological Services, Updated 2008 Climate of Brunei Darussalam.* Available at: http://www.bruneiweather.com.bn

[B16] Davies S. Becker P. (1996). Floristic composition and stand structure of mixed dipterocarp and heath forests in Brunei Darussalam. *J. Trop. For. Sci.* 8 542–569.

[B17] De Beeck M. O. Ruytinx J. Smits M. M. Vangronsveld J. Colpaert J. V. Rineau F. (2015). Belowground fungal communities in pioneer Scots pine stands growing on heavy metal polluted and non-polluted soils. *Soil Biol. Biochem.* 86 58–66. 10.1016/j.soilbio.2015.03.007

[B18] Din H. Metali F. Sukri R. S. (2015). Tree diversity and community composition of the Tutong white sands, Brunei Darussalam: a rare tropical heath forest ecosystem. *Int. J. Ecol.* 2015:2015 10.1155/2015/807876

[B19] Edgar R. C. (2010). Search and clustering orders of magnitude faster than BLAST. *Bioinformatics* 26 2460–2461. 10.1093/bioinformatics/btq461 20709691

[B20] Edgar R. C. Haas B. J. Clemente J. C. Quince C. Knight R. (2011). UCHIME improves sensitivity and speed of chimera detection. *Bioinformatics* 27 2194–2200. 10.1093/bioinformatics/btr381 21700674 PMC3150044

[B21] Fierer N. Jackson R. B. (2006). The diversity and biogeography of soil bacterial communities. *Proc. Natl. Acad. Sci. U.S.A.* 103 626–631. 10.1073/pnas.0507535103 16407148 PMC1334650

[B22] Frankland J. C. (1998). Fungal succession—unravelling the unpredictable. *Mycol. Res.* 102 1–15. 10.1017/S0953756297005364

[B23] Gao C. Shi N. N. Liu Y. X. Peay K. G. Zheng Y. Ding Q. (2013). Host plant genus-level diversity is the best predictor of ectomycorrhizal fungal diversity in a Chinese subtropical forest. *Mol. Ecol.* 22 3403–3414. 10.1111/mec.12297 24624421

[B24] Gardes M. Bruns T. D. (1993). ITS primers with enhanced specificity for basidiomycetes-application to the identification of mycorrhizae and rusts. *Mol. Ecol.* 2 113–118. 10.1111/j.1365-294X.1993.tb00005.x 8180733

[B25] Herlemann D. P. Labrenz M. Jürgens K. Bertilsson S. Waniek J. J. Andersson A. F. (2011). Transitions in bacterial communities along the 2000 km salinity gradient of the Baltic Sea. *ISME J.* 5 1571–1579. 10.1038/ismej.2011.41 21472016 PMC3176514

[B26] Jesus E. D. Marsh T. L. Tiedje J. M. Moreira F. M. D. (2009). Changes in land use alter the structure of bacterial communities in Western Amazon soils. *ISME J.* 3 1004–1011. 10.1038/ismej.2009.47 19440233

[B27] Jing X. Sanders N. J. Shi Y. Chu H. Classen A. T. Zhao K. (2015). The links between ecosystem multifunctionality and above-and belowground biodiversity are mediated by climate. *Nat. Commun.* 6:8159 10.1038/ncomms9159 PMC4569729 26328906

[B28] Jones R. T. Robeson M. S. Lauber C. L. Hamady M. Knight R. Fierer N. (2009). A comprehensive survey of soil acidobacterial diversity using pyrosequencing and clone library analyses. *ISME J.* 3 442–453. 10.1038/ismej.2008.127 19129864 PMC2997719

[B29] Kanokratana P. Uengwetwanit T. Rattanachomsri U. Bunterngsook B. Nimchua T. Tangphatsornruang S. (2011). Insights into the phylogeny and metabolic potential of a primary tropical peat swamp forest microbial community by metagenomic analysis. *Microb. Ecol.* 61 518–528. 10.1007/s00248-010-9766-7 21057783

[B30] Kerfahi D. Tripathi B. M. Lee J. Edwards D. P. Adams J. M. (2014). The impact of selective-logging and forest clearance for oil palm on fungal communities in Borneo. *PLoS ONE* 9:e111525 10.1371/journal.pone.0111525 PMC4236049 25405609

[B31] Kim O. S. Cho Y. J. Lee K. Yoon S. H. Kim M. Na H. (2012). Introducing EzTaxon-e: a prokaryotic 16S rRNA gene sequence database with phylotypes that represent uncultured species. *Int. J. Syst. Evol. Microbiol.* 62 716–721. 10.1099/ijs.0.038075-0 22140171

[B32] Lauber C. L. Hamady M. Knight R. Fierer N. (2009). Pyrosequencing-based assessment of soil pH as a predictor of soil bacterial community structure at the continental scale. *Appl. Environ. Microbiol.* 75 5111–5120. 10.1128/AEM.00335-09 19502440 PMC2725504

[B33] Lee-Cruz L. Edwards D. P. Tripathi B. M. Adams J. M. (2013). Impact of logging and forest conversion to oil palm plantations on soil bacterial communities in Borneo. *Appl. Environ. Microbiol.* 79 7290–7297. 10.1128/AEM.02541-13 24056463 PMC3837752

[B34] Leff J. W. Jones S. E. Prober S. M. Barberán A. Borer E. T. Firn J. L. (2015). Consistent responses of soil microbial communities to elevated nutrient inputs in grasslands across the globe. *Proc. Natl. Acad. Sci. U.S.A.* 112 10967–10972. 10.1073/pnas.1508382112 26283343 PMC4568213

[B35] Lozupone C. Lladser M. E. Knights D. Stombaugh J. Knight R. (2011). UniFrac: an effective distance metric for microbial community comparison. *ISME J.* 5 169–172. 10.1038/ismej.2010.133 20827291 PMC3105689

[B36] MacKinnon K. (1996). *The Ecology of Kalimantan.* Oxford: Oxford University Press.

[B37] Masella A. P. Bartram A. K. Truszkowski J. M. Brown D. G. Neufeld J. D. (2012). PANDAseq: paired-end assembler for illumina sequences. *BMC Bioinformatics* 13:31 10.1186/1471-2105-13-31 PMC3471323 22333067

[B38] McGuire K. D’Angelo H. Brearley F. Gedallovich S. Babar N. Yang N. (2014). Responses of soil fungi to logging and oil palm agriculture in Southeast Asian tropical forests. *Microb. Ecol.* 69 733–747. 10.1007/s00248-014-0468-4 25149283

[B39] Meyer F. Paarmann D. D’Souza M. Olson R. Glass E. M. Kubal M. (2008). The metagenomics RAST server–a public resource for the automatic phylogenetic and functional analysis of metagenomes. *BMC Bioinformatics* 9:386 10.1186/1471-2105-9-386 PMC2563014 18803844

[B40] Millard P. Singh B. (2010). Does grassland vegetation drive soil microbial diversity? *Nutr. Cycl. Agroecosys.* 88 147–158. 10.1007/s10705-009-9314-3

[B41] Miyashita N. T. Iwanaga H. Charles S. Diway B. Sabang J. Chong L. (2013). Soil bacterial community structure in five tropical forests in Malaysia and one temperate forest in Japan revealed by pyrosequencing analyses of 16S rRNA gene sequence variation. *Genes Genet. Syst.* 88 93–103. 10.1266/ggs.88.93 23832301

[B42] Moran J. A. Barker M. G. Moran A. J. Becker P. Ross S. M. (2000). A comparison of the soil water, nutrient status, and litterfall characteristics of tropical heath and mixed-dipterocarp forest sites in Brunei1. *Biotropica* 32 2–13. 10.1646/0006-3606(2000)032[0002:ACOTSW]2.0.CO;2

[B43] Nacke H. Fischer C. Thürmer A. Meinicke P. Daniel R. (2014). Land use type significantly affects microbial gene transcription in soil. *Microb. Ecol.* 67 919–930. 10.1007/s00248-014-0377-6 24553913

[B44] Oksanen J. Kindt R. Legendre P. O’Hara B. Stevens M. Oksanen M. (2007). *The Vegan Package. Community Ecology Package 2.0-7.*

[B45] Osono T. (2007). Ecology of ligninolytic fungi associated with leaf litter decomposition. *Ecol. Res.* 22 955–974. 10.1007/s11284-007-0390-z

[B46] Pacchioni R. G. Carvalho F. M. Thompson C. E. Faustino A. L. Nicolini F. Pereira T. S. (2014). Taxonomic and functional profiles of soil samples from Atlantic forest and Caatinga biomes in northeastern Brazil. *Microbiologyopen* 3 299–315. 10.1002/mbo3.169 24706600 PMC4082704

[B47] Page S. Rieley J. Wüst R. (2006). Lowland tropical peatlands of Southeast Asia. *Dev Earth Surf. Proc.* 9 145–172. 10.1016/S0928-2025(06)09007-9

[B48] Peay K. G. Kennedy P. G. Davies S. J. Tan S. Bruns T. D. (2010). Potential link between plant and fungal distributions in a dipterocarp rainforest: community and phylogenetic structure of tropical ectomycorrhizal fungi across a plant and soil ecotone. *New Phytol.* 185 529–542. 10.1111/j.1469-8137.2009.03075.x 19878464

[B49] Prosser J. I. (2012). Ecosystem processes and interactions in a morass of diversity. *FEMS Microbiol. Ecol.* 81 507–519. 10.1111/j.1574-6941.2012.01435.x 22715974

[B50] Pupin B. Nahas E. (2014). Microbial populations and activities of mangrove, restinga and Atlantic forest soils from Cardoso Island, Brazil. *J. Appl. Microbiol.* 116 851–864. 10.1111/jam.12413 24314121

[B51] Rodrigues J. L. Pellizari V. H. Mueller R. Baek K. Jesus Eda C. Paula F. S. (2013). Conversion of the Amazon rainforest to agriculture results in biotic homogenization of soil bacterial communities. *Proc. Natl. Acad. Sci. U.S.A.* 110 988–993. 10.1073/pnas.1220608110 23271810 PMC3549139

[B52] Russo S. E. Legge R. Weber K. A. Brodie E. L. Goldfarb K. C. Benson A. K. (2012). Bacterial community structure of contrasting soils underlying Bornean rain forests: inferences from microarray and next-generation sequencing methods. *Soil Biol. Biochem.* 55 48–59. 10.1016/j.soilbio.2012.05.021

[B53] Satrio A. E. Gandaseca S. Ahmed O. H. Nik Muhamad A. (2009). Effect of precipitation fluctuation on soil carbon storage of a tropical peat swamp forest. *Am. J. Appl. Sci.* 6 1484–1488. 10.3844/ajassp.2009.1484.1488

[B54] Schloss P. D. Westcott S. L. Ryabin T. Hall J. R. Hartmann M. Hollister E. B. (2009). Introducing mothur: open-source, platform-independent, community-supported software for describing and comparing microbial communities. *Appl. Environ. Microbiol.* 75 7537–7541. 10.1128/AEM.01541-09 19801464 PMC2786419

[B55] Slik J. W. F. Poulsen A. Ashton P. Cannon C. Eichhorn K. Kartawinata K. (2003). A floristic analysis of the lowland dipterocarp forests of Borneo. *J. Biogeogr.* 30 1517–1531. 10.1046/j.1365-2699.2003.00967.x

[B56] Slik J. W. F. Raes N. Aiba S. I. Brearley F. Q. Cannon C. H. Meijaard E. (2009). Environmental correlates for tropical tree diversity and distribution patterns in Borneo. *Divers. Distrib.* 15 523–532. 10.1111/j.1472-4642.2009.00557.x

[B57] Sukri R. S. Wahab R. A. Salim K. A. Burslem D. F. (2012). Habitat associations and community structure of dipterocarps in response to environment and soil conditions in Brunei Darussalam, northwest Borneo. *Biotropica* 44 595–605. 10.1111/j.1744-7429.2011.00837.x

[B58] Tedersoo L. May T. W. Smith M. E. (2010). Ectomycorrhizal lifestyle in fungi: global diversity, distribution, and evolution of phylogenetic lineages. *Mycorrhiza* 20 217–263. 10.1007/s00572-009-0274-x 20191371

[B59] Tripathi B. M. Kim M. Lai-Hoe A. Shukor N. A. A. Rahim R. A. Go R. (2013). pH dominates variation in tropical soil archaeal diversity and community structure. *FEMS Microbiol. Ecol.* 86 303–311. 10.1111/1574-6941.12163 23773164

[B60] Tripathi B. M. Kim M. Singh D. Lee-Cruz L. Lai-Hoe A. Ainuddin A. (2012). Tropical soil bacterial communities in Malaysia: pH dominates in the equatorial tropics too. *Microb. Ecol.* 64 474–484. 10.1007/s00248-012-0028-8 22395784

[B61] Tripathi B. M. Lee-Cruz L. Kim M. Singh D. Go R. Shukor N. A. (2014). Spatial scaling effects on soil bacterial communities in Malaysian tropical forests. *Microb. Ecol.* 68 247–258. 10.1007/s00248-014-0404-7 24658414

[B62] White T. J. Bruns T. Lee S. Taylor J. (1990). “Amplification and direct sequencing of fungal ribosomal RNA genes for phylogenetics,” in *PCR Protocols: A Guide to Methods and Applications*, eds Innis M. A. Gelfand D. H. Sninsky J. J. White T. J. (San Diego, CA: Academic Press), 315–322.

[B63] Whitmore T. (1984). *Tropical Rain Forests of the Far East*, 2nd Edn Oxford: Oxford University Press.

