# Supplementary material for: Distinctive Tropical Forest Variants Have Unique Soil Microbial Communities, But Not Always Low Microbial Diversity
Source: Front Microbiol. 2016 Apr 5;7:376. doi: 10.3389/fmicb.2016.00376 (PMC4820907; doi:10.3389/fmicb.2016.00376)
Supplement: Supplementary file 1 [file Table_1.DOCX]

***Supplementary Material***

**Distinctive tropical forest variants have unique soil microbial communities, but not always low microbial diversity**

Binu M. Tripathi^1^, Woojin Song^1,2^, [J. W. Ferry Slik](http://www.pnas.org/search?author1=J.+W.+Ferry+Slik&sortspec=date&submit=Submit)^3^, Rahayu S. Sukri^3^, Salwana Jaafar^3^, Ke Dong^1^, Jonathan M. Adams^1*^

^1^Department of Biological Science, College of Natural Sciences, Seoul National University, Seoul, Republic of Korea.

^2^Seoul Zoo, Seoul Grand Park, Daegongwongwangjang-ro, Gwacheon-si, Seoul, Republic of Korea.

^3^Faculty of Science, Universiti Brunei Darussalam, Jln Tungku Link, Gadong, Brunei Darussalam.

***Correspondence:** Jonathan M. Adams, [geograph.ecol@gmail.com](mailto:geograph.ecol@gmail.com)

**Table S1.** Comparison of relative abundance (means±SD) of dominant EcM genera among forest types.

| **Dominant**  **EcM genera** | **MDF primary**  **forest** | **MDF secondary forest** | **White sand heath forest** | **Inland heath forest** | **Peat swamp forest** |
| --- | --- | --- | --- | --- | --- |
| *Russula* | 22.96±17.76 a | 8.36±17.56 b | 0.03±0.03 c | 6.60±11.43 b | 0.19±0.42 c |
| *Amanita* | 1.12±1.41 ab | 2.39±5.81 ab | 0.003±0.008 c | 2.56±2.49 a | 0.02±0.05 bc |
| *Thelephora* | 0.00±0.00 a | 1.43±4.29 a | 0.00±.0.00 a | 0.00±0.00 a | 0.00±0.00 a |
| *Tomentella* | 0.21±0.31 a | 0.43±0.59 a | 0.00±0.00 b | 0.02±0.05 b | 0.68±0.79 a |

Different letters represent means that were significant different (*P <* 0.05) based on pairwise Wilcox test followed by Benjamini–Hochberg correction for multiple comparisons.

**Supplementary figure legends**

**Figure S1.** Principal components analysis of sampling points based on soil properties only. Vectors represent the direction of increase for a given variable and its length indicates the strength of the correlation between the variable and the ordination scores. GWC= gravimetric water content and OM = organic matter content.

**Figure S2.** UPGMA cluster analysis comparing the bacterial and fungal communities in samples of five different forest types.

**Figure S3.** Nonmetric multidimensional scaling plot of bacterial communities based on pairwise unweighted UniFrac distances. A vector overlay of the significantly correlated variables is shown on the plot. GWC = gravimetric water content and OM = organic matter content.
