# Supplementary figures and images for: Distinctive Tropical Forest Variants Have Unique Soil Microbial Communities, But Not Always Low Microbial Diversity
Source: Front Microbiol. 2016 Apr 5;7:376. doi: 10.3389/fmicb.2016.00376 (PMC4820907; doi:10.3389/fmicb.2016.00376)

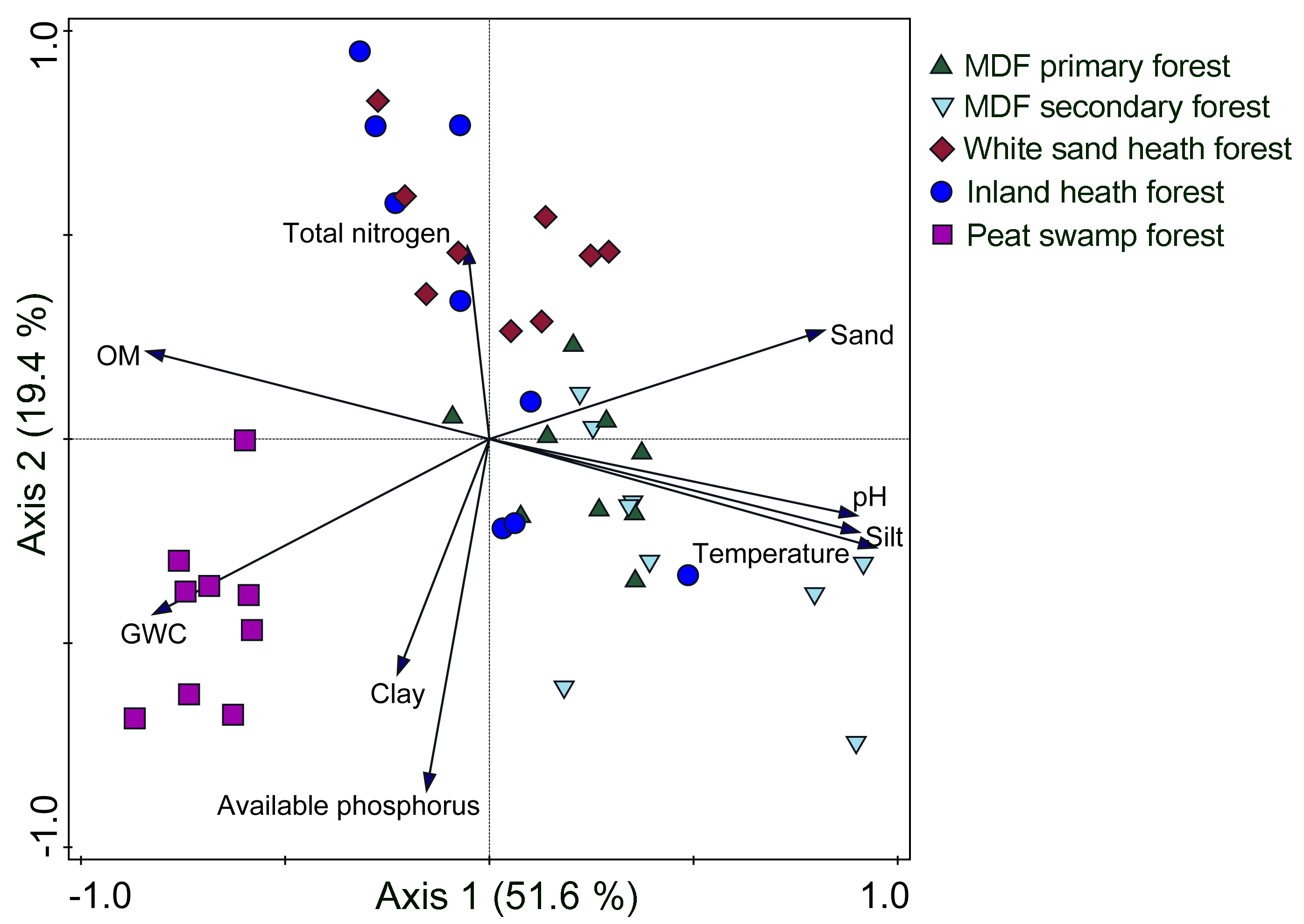

Supplement: Supplementary file 2 [file Image_1.TIF]

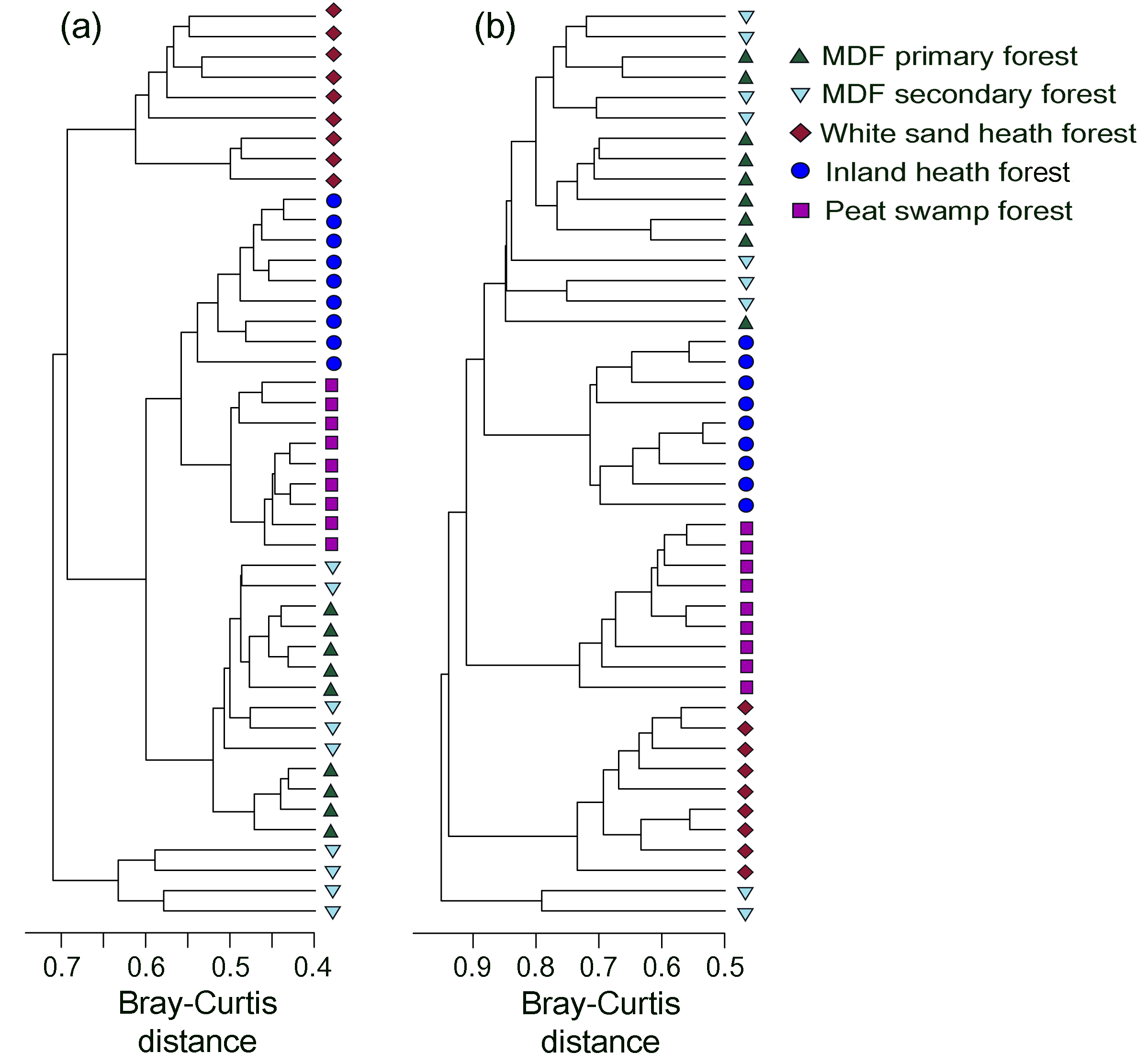

Supplement: Supplementary file 3 [file Image_2.TIF]

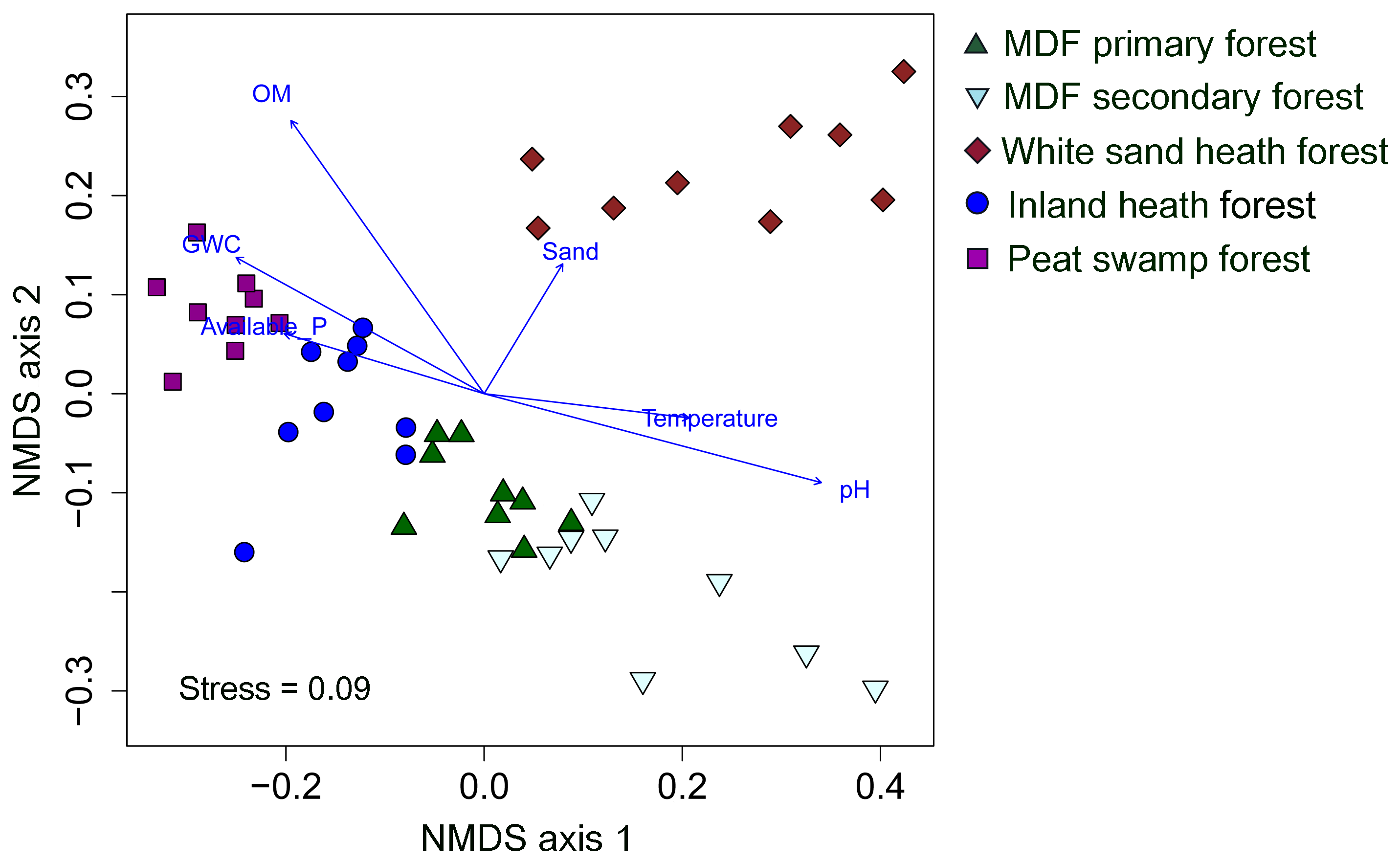

Supplement: Supplementary file 4 [file Image_3.TIF]
